# AI for interpreting screening mammograms: implications for missed cancer in double reading practices and challenging-to-locate lesions

**DOI:** 10.1038/s41598-024-62324-4

**Published:** 2024-05-24

**Authors:** Zhengqiang Jiang, Ziba Gandomkar, Phuong Dung (Yun) Trieu, Seyedamir Tavakoli Taba, Melissa L. Barron, Sarah J. Lewis

**Affiliations:** 1 Discipline of Medical Imaging Sciences, School of Health Sciences, Faculty of Medicine and Health, The University of Sydney, Sydney, Australia; 2 School of Health Sciences, Western Sydney University, Campbelltown, Australia

**Keywords:** Artificial intelligence, Concordance correlation coefficient, Mammography, Transfer learning, Saliency maps, Diagnosis, Cancer, Health care

## Abstract

Although the value of adding AI as a surrogate second reader in various scenarios has been investigated, it is unknown whether implementing an AI tool within double reading practice would capture additional subtle cancers missed by both radiologists who independently assessed the mammograms. This paper assesses the effectiveness of two state-of-the-art Artificial Intelligence (AI) models in detecting retrospectively-identified missed cancers within a screening program employing double reading practices. The study also explores the agreement between AI and radiologists in locating the lesions, considering various levels of concordance among the radiologists in locating the lesions. The Globally-aware Multiple Instance Classifier (GMIC) and Global–Local Activation Maps (GLAM) models were fine-tuned for our dataset. We evaluated the sensitivity of both models on missed cancers retrospectively identified by a panel of three radiologists who reviewed prior examinations of 729 cancer cases detected in a screening program with double reading practice. Two of these experts annotated the lesions, and based on their concordance levels, cases were categorized as 'almost perfect,' 'substantial,' 'moderate,' and 'poor.' We employed Similarity or Histogram Intersection (SIM) and Kullback–Leibler Divergence (KLD) metrics to compare saliency maps of malignant cases from the AI model with annotations from radiologists in each category. In total, 24.82% of cancers were labeled as “missed.” The performance of GMIC and GLAM on the missed cancer cases was 82.98% and 79.79%, respectively, while for the true screen-detected cancers, the performances were 89.54% and 87.25%, respectively (*p*-values for the difference in sensitivity < 0.05). As anticipated, SIM and KLD from saliency maps were best in ‘almost perfect,’ followed by ‘substantial,’ ‘moderate,’ and ‘poor.’ Both GMIC and GLAM (*p*-values < 0.05) exhibited greater sensitivity at higher concordance. Even in a screening program with independent double reading, adding AI could potentially identify missed cancers. However, the challenging-to-locate lesions for radiologists impose a similar challenge for AI.

## Introduction

Breast cancer is the most common cancer among women, with approximately 2.3 million new cases diagnosed worldwide in 2020, and one of the leading causes of cancer deaths, with an estimated 685,000 deaths per year^1^. The early detection of breast cancer can help to improve survival rates and treatment choices, with the five-year survival rate ranging from 90% survival for breast cancer diagnosed at Stage 1, to 35% for Stage 4^2^. Mammography is a mainstream breast screening imaging technique for cancer diagnosis worldwide. A screening mammogram generally consists of x-ray imaging with 2 standard views of each breast: a mediolateral oblique (MLO) view taken with compression from the lateral side of the breast with an angled tube and a craniocaudal (CC) view taken with compression from superior to inferior with a straight tube. In the screening scenario through the national program BreastScreen Australia (BSA), there are two possible pathways. Women may be recalled for further imaging or examinations if there is suspicion of a cancer. For cases where the radiological interpretation is that no cancer is present, women will generally receive a recommendation to ‘return to screening’ at a defined interval, usually every two years. For women who are recalled, additional tests such as further X-ray imaging, ultrasound and biopsy may be required through an assessment clinic. A doctor will explain the examination results and follow-up procedures for positive results.

Interpretation of the screening mammography is a challenging task. A recent large-scale study, which included 418,041 women and 110 radiologists, showed that overall sensitivity of radiologists was 73% which means about 27% of cancers were missed or not acted on^3^. Although these radiologists interpreted more than 5000 annual screening mammograms and were considered high-volume screen readers, they still exhibited relatively low sensitivity. When double reading and consensus discussion is practiced, the overall sensitivity of the entire process reaches 85%^3^. Although some diagnostic errors are inevitable when perceptual tasks are practised, the error rates remain unacceptably high. As a technique to mitigate these errors, various computerized tools have been developed over the past decades to assist radiologists with mixed results and clinical uptake.

Artificial Intelligence (AI) has been widely used in visual object detection and/or recognition, providing promising results in the last decade^4–9^. The application of AI with deep learning in medical imaging analysis for cancer diagnosis has attracted strong interest from researchers, and practitioners in radiology. High quality medical images with accurate annotations are required to train a reliable and effective AI model for cancer diagnosis. Training AI models for clinical use also requires large datasets. Once trained, these models can potentially alleviate workload pressures on radiologists/physicians and improve cancer diagnosis efficiency. AI has been deployed for medical image analysis in abnormal lesion detection in the lung^10^, liver^11^, brain^12^, and breast^13,14^. A recent meta-analysis^15^ concluded that standalone AI performed as well as or better than radiologists in screening digital mammography.

Retrospective studies have suggested that AI tools can assist radiologists in reducing the number of missed cancers^16^. In cases where mammographic examinations undergo double reading practices, with two readers independently interpreting the images, such as with BSA, the incidence of missed cancers substantially decreases. However, based on these studies, it remains unknown whether AI can help identify subtle cancers beyond what is detected by two radiologists. There is only a single prospective study^17^ that explored triple reading by two radiologists plus AI in the context of Swedish breast cancer screening. Based on the cancer detection rate, the study concluded that triple reading was deemed superior to double reading with two radiologists. However, no previous study has compared AI's sensitivity on a cohort of missed cancers from a double reading scenario against its performance for true screen-detected cancers. It is unknown whether these cases pose more significant challenges for AI and if AI tools’ sensitivity values are significantly for these missed cases. Further investigation is needed to fully understand the capabilities of AI tools in the double reading setting and their potential to enhance program’s sensitivity. Particularly, if AI tools are intended to be used as part of the current practice without major changes (i.e., replacing one of the readers with AI), the added benefit of AI tools in double reading practice should be thoroughly investigated.

In addition to providing a diagnosis for a mammographic examination at the case level, many AI tools offer a saliency map or annotated image that aids radiologists in identifying and localizing suspicious areas or lesions detected by the AI tools. These annotated images or maps can also assist radiologists in reporting important characteristics of the lesion, such as shape, margin, and tumor size on mammograms^18^. However, even experienced radiologists may differ in their interpretation of lesion locations. It remains unclear whether lesions that are challenging to annotate for human observers are also difficult for AI tools to localize and detect. Addressing this question would provide a better understanding of the potential applications of AI in this context to reduce inter-reader variability.

This paper investigates the performance of two state-of-the-art AI models, Globally-aware Multiple Instance Classifier (GMIC)^13^ and Global–Local Activation Maps (GLAM)^14^. The main objectives of this study are as follows: (1) to assess whether these AI tools can identify retrospectively-identified missed cancers in a screening with double-reader practice and determine if these missed cancers pose a greater challenge for the AI tools in terms of detection; (2) to explore the concordance between radiologists in annotating lesion locations and the agreement between radiologists' annotations and the saliency maps generated by AI; and (3) to investigate whether lesions that are challenging to annotate are also difficult for the AI to detect. We compared the saliency maps from GLAM and GMIC with annotations from two radiologists in four concordance levels using two most common metrics: similarity or histogram intersection (SIM) and Kullback–Leibler divergence (KLD)^19^.

Transfer learning of the two AI models was conducted on 1712 mammographic cases (856 proven cancer and 856 normal or benign cases) derived from an Australian clinical research database linked to BreastScreen Australia. The cases were collected from multiple screening centers, from diverse cultural and ethnic populations and with different imaging vendors. The application of transfer learning of these two AI models on our image sets was also tested and an optimal protocol was developed. Transfer learning^20^ is one machine learning method, which involves training a pre-existing AI model on available data and can train effective AI models with limited training data. Transfer learning has been very widely used in development of AI tools breast cancer detection^21,22^. Here, we explored its value for transferring a model trained based on another dataset from another screening setting to our cohort. The diagnostic accuracy of two AI models were evaluated through specificity and sensitivity in mammograms in four concordance levels.

## Materials and methods

### Datasets

#### Dataset 1: retrospectively reviewed *cancer* cases to determine missed cancers in double-reading practice.

Two breast radiologists with an annual workload of over 5000 mammograms and 15 + years of experience in reading mammograms categorized 729 mammographic cases and assigned them into “missed” cancers, “prior-vis” cancers and “prior-invis” by comparing the current mammographic examination with the prior ones. In case of disagreement in categorization, a third radiologist (with 15 + years of experience in breast imaging) acted as a senior arbitrator. All cases were confirmed to be cancer cases through histopathological report. For these 729 cases, all had at least one prior screening examination, which was reported as normal by two BSA radiologists in the routine breast cancer screening program. A “missed” cancer was defined as being visible on the prior mammographic examination in our experiment, but not seen during the screening and not recalled or acted on. For “prior-vis” case classification, non-actionable cancer signs were visible on the prior examinations by the two radiologists in our present study with the benefit of the histopathological report of the current cancer case. For a “prior-invis” case, the two radiologists in this experiment could not reasonably see the cancer on the prior images even with the benefit of the histopathological report of current cancer images and therefore it was considered that this is not an error by the original two radiologists in the routine double reading process. There were no known interval cases in Dataset 1.

#### Dataset 2: annotated screening mammograms

The annotations for 856 malignant cases, including 729 cases from Dataset 1 and an additional 127 cancer cases, were obtained from two highly experienced breast radiologists specializing in screening mammograms, each with over 20 years of experience in breast imaging. Each case had four mammograms on the left and right breast: left and right MLO and CC views. The radiologists were informed that all cases contain at least one biopsy-proven cancer, and each breast may have more than one cancer signs. The radiologists were instructed to draw an unfilled box which enclosed tightly the regions of interest with cancer signs on abnormal mammographic images but were not aware of each other’s annotations. The lesion areas marked by the radiologists were confirmed by the pathological reports. Pathological reports, retrieved from the screening archive, were also available to the radiologists. This information provided the side of the malignancy and histopathological characteristics (e.g., invasive (312 cases) or ductal carcinoma in-situ (503 cases), breast cancer grade, immunohistochemistry quantification, and breast cancer stage). The cancer sizes were classified as three groups: T1 (< = 2 cm) with 579 cases, T2(> 2 cm & < 5 cm) with 208 cases, and T3(> = 5 cm) with 69 cases according to the American Cancer Society. The overlapped annotations between two radiologists were defined as two boxes with a size of overlapping area greater than 0. The overlapping annotations were determined by Intersection over Union (IoU)^23^ score between the boxes drawn by the two radiologists. Lin’s concordance correlation coefficient (CCC)^24^ was computed based on four corner points of overlapped annotations from two radiologists. The values of Lin’s CCC are in the range [-1, 1], with a large value indicating strong agreement between 2 radiologists’ annotations and a small value indicating strong disagreement.

A challenging-to-localise cancer is defined as a case for which the annotations from radiologists did not significantly overlap (IoU > 0.95). We evaluated the values of Lin’s CCC between the two radiologists’ annotations according to the interpretation guide described in McBride's paper^25^. McBride interpreted values of Lin’s CCC in four concordance levels: ‘almost perfect’ (values greater than 0.99), ‘substantial’ (values between 0.95 and 0.99), ‘moderate’ (values between 0.9 and 0.95) and ‘poor’ (values less than 0.9).

### Artificial intelligence models

The GLAM and GMIC models were used as our AI models. For the completeness of this study, a brief review of these two AI models is provided in this subsection. These models were selected because they both could produce saliency maps and incorporate the global context into their decision-making process. They are both among the highest-performing AI models in the literature and are publicly available. In previous published work^14^, GMIC outperformed GLAM in terms of classification performance, while GLAM showed superior performance in terms of segmentation; hence, we included both models in this current study for testing. However, both models were trained using images from different screening setting. To transfer these models to our dataset, we used a data set of 1712 mammographic cases for transfer learning. The dataset included 856 biopsy-proven cancer cases from Dataset 2 and 856 normal cases, matched based on age and mammography units used for the image acquisition*.* We conducted a fourfold cross-validation to train and validate the two AI models with transfer learning.

#### Globally-aware multiple instance classifier (GMIC)

The GMIC model combines global and local features on mammographic images for breast screening interpretation. Firstly, the GMIC classifier used a ResNet-22 network^26^ to extract global feature maps as a way of trying to mimic the global impression that radiologists use while reading through an entire image. Each global feature map was convolved with a 1 × 1 filter and applied a sigmoid operation, which then used top *t*% pooling to generate benign and malignant feature maps. Image patches were selected from the two feature maps by searching the largest average intensity of the patches. Similarly, extracting feature maps from image patches mimicked the way radiologists would concentrate on suspicious lesion areas. Feature maps of image patches were fine-tuned using a ResNet-50 network^26^ and then weighted via gated attention mechanism. The final process combined the global feature map and attention-weighted representation of image patches to predict image classification. All the mammographic images for GMIC were resized in 1920 × 2944 pixels using bilinear interpolation^27^. The parameter *t* for top *t*% pooling was set as 6. For the GMIC^13^ model, we used the source codes published by the authors on the GitHub at https://github.com/nyukat/GMIC.git.

#### Global–local activation maps (GLAM)

GLAM has extended the global feature module from GIMC to generate multi-scale feature maps on mammograms. Such a method consisted of three main stages. The first stage used a convolutional neural network (CNN) similar to ResNet^26^ to extract a global saliency map and predict classification for each mammographic image. Saliency maps at different layers of CNN were computed with convolutional operations and Sigmoid functions to generate cross-entropy loss and then concatenated to generate potential segmentation patches in multiple scales. The second stage generated a set of patches from the feature map based on the local maximum of average intensity. In the last stage, a ResNet-34 network^26^ was applied to the image patches to fine-tune the saliency map, which was then aligned to the position of the corresponding mammographic image. All saliency maps of patches were combined to give output class prediction of a mammographic image using concatenation-based and attention-based aggregations and resizing was performed as described above for GMIC. The sizes of saliency maps in 3 scales for the global module were set as 184 × 120, 92 × 60, and 46 × 30, respectively. For the GLAM model, we used the source codes published by the authors on the GitHub at https://github.com/nyukat/GLAM.git.

### Transfer learning on our dataset

Transfer learning of pre-trained models of GMIC and GLAM methods was conducted on mammographic images in our dataset 2. Both the pre-trained models were trained on The NYU Breast Cancer Screening (NYUBCS) Dataset^28^. While the examinations in NYUBCS dataset were acquired using Mammomat Inspiration, Mammomat Novation DR (Siemens), Lorad Selenia and Selenia Dimensions (Hologic), our dataset also included images from other manufacturers including Fuji, Sectra, GE Healthcare and Philips Healthcare. Hence, we undertook fine-tuning of the GMIC and GLAM models on our dataset. The model training was an iterative process that compared the accuracy of the models in the current epoch with that in the previous epoch. The number of epochs was set as 100. The training was concluded when the callback in the validation process did not improve the accuracy of the models with 3 epochs. Early stopping at Epoch 83 was encountered during the training process in this study.

For the uniformity in transfer learning of GMIC model, we resized all the mammographic images to 2944 × 1920 pixels. To reduce training time of the model by 5.7%, we tightly cropped each mammographic image to include breast region and removed background information using connected-component labelling algorithm and morphological operations. The model outputted the malignant probabilities and saliency maps with the same sizes of inputting images. As suggested in the original papers, the RCC and RMLO mammographic images were flipped and marked so that the left and right breasts were present on the left side of all images. We trained the transfer learning model using the Adam optimization algorithm^29^ with a learning rate of 10^−5^. The loss function used the binary cross-entropy^30^. Both the width and the height of the global saliency map were set as 256. Setting the number *K* of patches from the global saliency map for local module was relatively complex because the classification performance improved with an increase in *K* but fluctuated when *K* > 3. Thus, *K* was set as 3 in this study.

For the transfer learning of GLAM model, the processes of resizing, cropping and flipping images were similar to GMIC model. The transfer learning model of GLAM also used Adam optimization algorithm^30^ with a learning rate of 10^−5^ and the binary cross-entropy loss function^30^. Both the width and the height of the global saliency map were set as 512. The model fed mammographic images into a ResNet-22 network and returned 256 intermediate feature maps with 46 × 30 size. The larger number of patches selected for the local module improve the classification performance for GLAM model. Therefore, to balance the computational time, the number of patches was set as 6.

### Evaluation metrics

#### Specificity and sensitivity metric

Specificity and sensitivity metrics were used to evaluate the performance of the AI models with and without transfer learning (the pre-trained vs transfer learning modes). We also evaluated the sensitivity of the two AI models on ‘missed’ cancers, ‘prior-vis’ and ‘prior-invis’ cancers among four concordance levels. Two common metrics (SIM and KLD) were used to compare the saliency maps of the two AI methods and the two radiologists’ annotations.

#### Similarity or histogram intersection metric

Similarity or Histogram Intersection (SIM)^31^ was deployed to compute the similarity between two histograms of pixel values of malignant saliency maps from GLAM and GIMC on mammographic images. Given the histograms (${H}^{1}, {H}^{2}$) of pixel values of malignant saliency maps from GLAM and GMIC on mammographic images, the computation of SIM is formulated as follows:

$$SIM\left( {H^{1} ,{ }H^{2} } \right) = \mathop \sum \limits_{i} {\text{min}}\left( {H_{i}^{1} ,H_{i}^{2} } \right)$$

where the term *i* is the bin index of the histogram. The histogram of pixel values of malignant saliency map was normalized to satisfy $\sum_{i}{H}_{i}^{1}=\sum_{i}{H}_{i}^{2}=1$. The values of SIM are in range [0, 1], where a large value indicates more overlap between histogram distributions of two malignant saliency maps.

#### Kullback–Leibler divergence metric

Kullback–Leibler Divergence (KLD)^32^ was used to compute the difference between two probability distribution of malignant saliency maps from GLAM and GIMC on mammographic images. Given the two-probability distribution (${D}^{1}, {D}^{2}$) of malignant saliency maps from GLAM and GMIC on mammographic images, the computation of KLD is formulated as follows:

$$KLD\left( {D^{1} ,D^{2} } \right) = \mathop \sum \limits_{i} D^{1} {\text{log}}\left( {\epsilon + \frac{{D^{1} }}{{\epsilon + D^{2} }}} \right)$$

where $\epsilon$ is a regularization constant and was set as 1e^−10^. The probability distribution of malignant saliency map was normalized to satisfy $\sum_{i}{D}_{i}^{1}=\sum_{i}{D}_{i}^{2}=1$. The values of KLD are in range of 0 to 1, where small values indicate closeness between the probability distributions of two malignant saliency maps. Saliency maps of both GLAM and GIMC models were compared with the annotations of two radiologists. The same saliency maps of these AI models were then compared with different concordance levels.

### Institutional review board

The study was approved by Human Ethics Research Committee of the University of Sydney (2019/1017). The research was conducted in accordance with the Declaration of Helsinki. All methods were performed in accordance with the relevant guidelines and regulations.

### Informed consent

Informed consent was obtained from all subjects involved in the study.

## Results

### Localization performance

**Figure 1:**
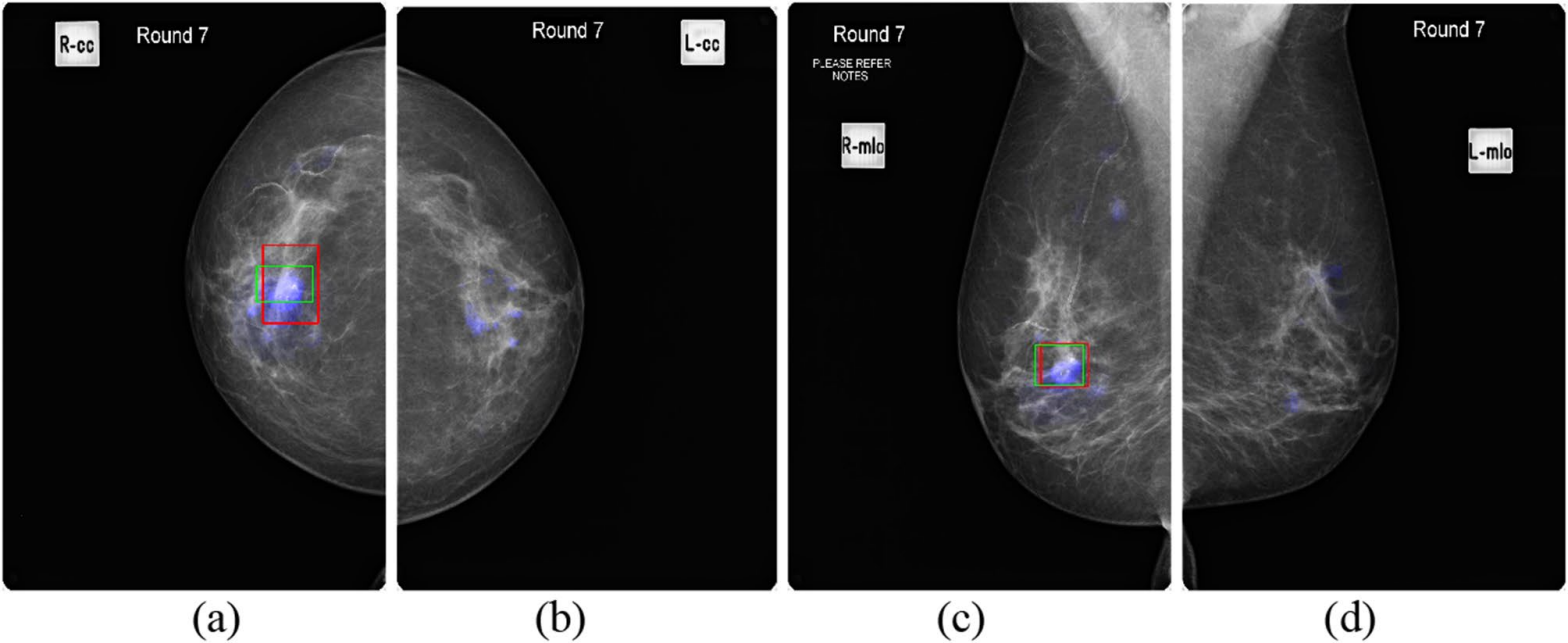
GLAM on mammography images in four views for Patient 1 with malignancy. (**a**) RCC view. (**b**) LCC view. (**c**) RMLO view. (**d**) LMLO view. (The cancer is at the upper outer quadrant of the right breast. Blue colour corresponds to the saliency map, red colour corresponds to the annotations of Radiologist A, and green colour corresponds to the annotations of Radiologist B).

**Figure 2:**
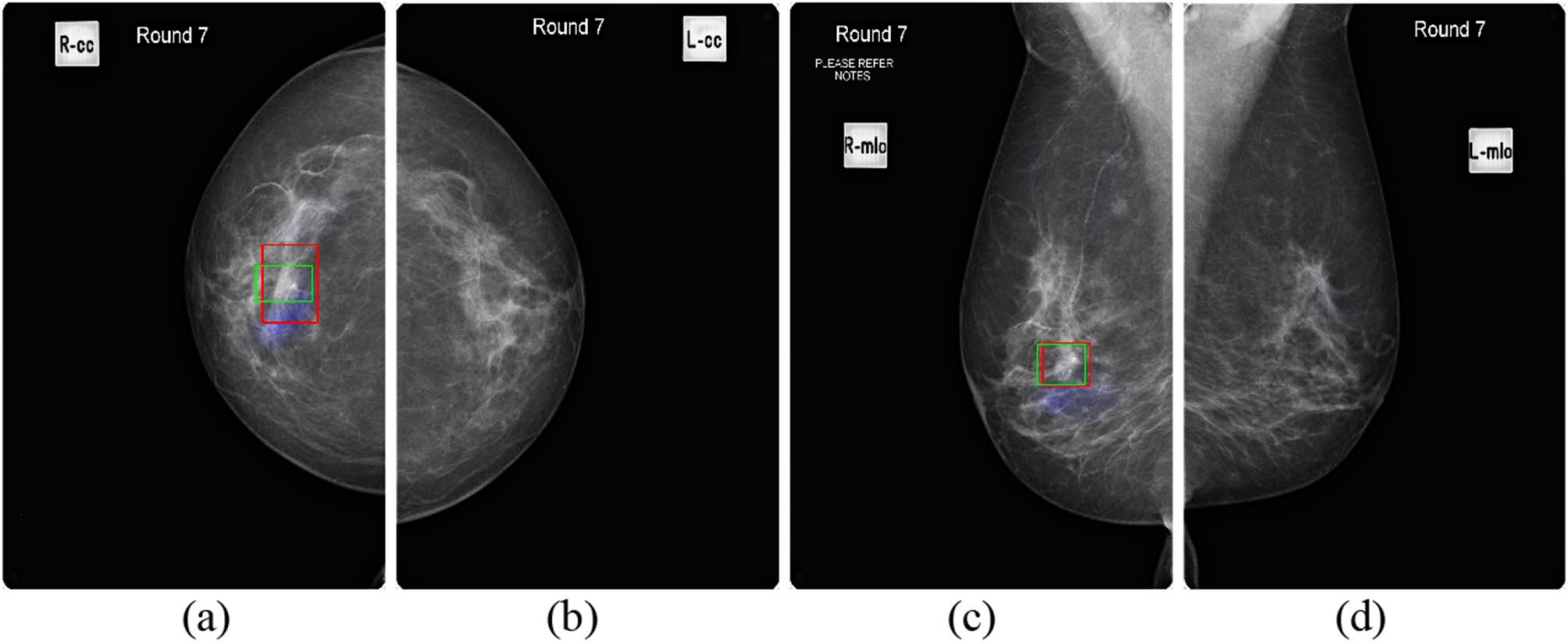
GMIC on mammography images in four views for Patient 1 with malignancy. (**a**) RCC view. (**b**) LCC view. (**c**) RMLO view. (**d**) LMLO view. (The cancer is at the upper outer quadrant of the right breast. Blue colour corresponds to the saliency map, red colour corresponds to the annotations of Radiologist A, and green colour corresponds to the annotations of Radiologist B).

**Figure 3:**
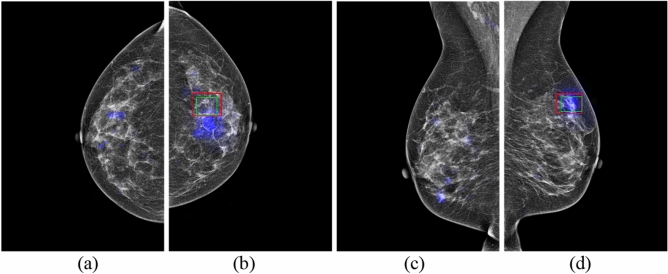
GLAM on mammography images in four views for Patient 2 with malignancy. (**a**) RCC view. (**b**) LCC view. (**c**) RMLO view. (**d**) LMLO view. (The cancer is at the upper outer quadrant of the left breast. Blue colour corresponds to the saliency map, red colour corresponds to the annotations of Radiologist A, and green colour corresponds to the annotations of Radiologist B).

**Figure 4:**
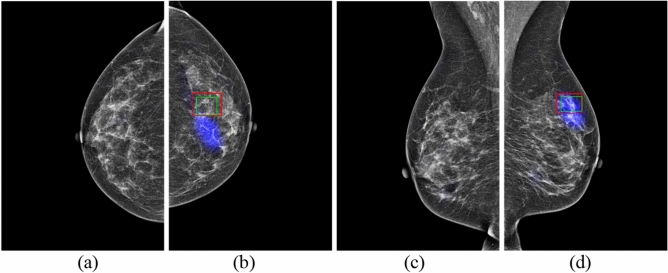
GMIC on mammography images in four views for Patient 2 with malignancy. (**a**) RCC view. (**b**) LCC view. (**c**) RMLO view. (**d**) LMLO view. (The cancer is at the upper outer quadrant of the left breast. Blue colour corresponds to the saliency map, red colour corresponds to the annotations of Radiologist A, and green colour corresponds to the annotations of Radiologist B).

**Figure 5:**
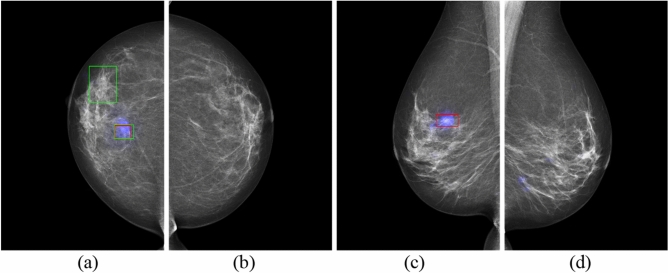
GLAM on mammography images in four views for Patient 3 with malignancy. (**a**) RCC view. (**b**) LCC view. (**c**) RMLO view. (**d**) LMLO view. (The cancer is located centrally in the right breast. Blue colour corresponds to the saliency map, red colour corresponds to the annotations of Radiologist A, and green colour corresponds to the annotations of Radiologist B).

**Figure 6:**
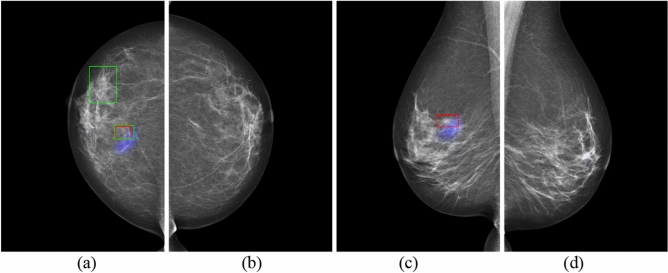
GMIC on mammography images in four views for Patient 3 with malignancy. (**a**) RCC view. (**b**) LCC view. (**c**) RMLO view. (**d**) LMLO view. (The cancer is located centrally in the right breast. Blue colour corresponds to the saliency map, red colour corresponds to the annotations of Radiologist A, and green colour corresponds to the annotations of Radiologist B).

**Figure 7:**
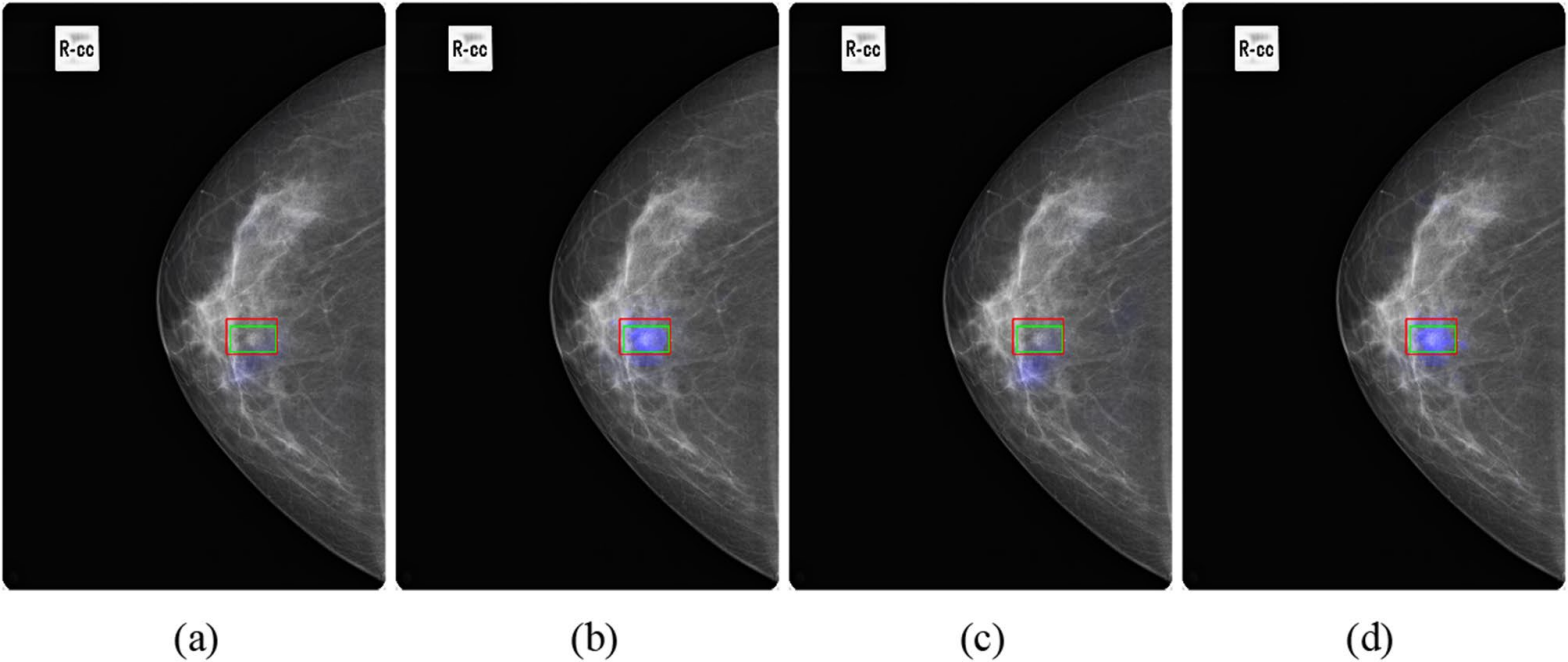
Results of GLAM and GMIC with and without transfer learning on our dataset. (**a**) GLAM only. (**b**) GLAM with transfer learning. (**c**) GMIC only. (**d**) GMIC with transfer learning. (Blue color corresponds to the saliency map, red color corresponds to the annotations of Radiologist A, and green color corresponds to the annotations of Radiologist B).

Saliency maps generated by the AI models and radiologists' annotations on mammographic images for three patients are presented in Figs. 1, 2, 3, 4, 5, 6 as exemplars. Figures 1 and 2 show the results for Patient 1 in four different views. Figures 3 and 4 show the corresponding results for Patient 2, while Figs. 5 and 6 are for Patient 3. A comparison of the results of GLAM only and GLAM with transfer learning, and GIMC only and GMIC with transfer learning are shown in Fig. 7. The saliency maps from GLAM and GMIC are shown in blue, while annotations in red are from Radiologist A and annotations in green from Radiologist B.

As shown in these figures, the saliency maps of GLAM matched better with the radiologists’ annotations than those of GMIC. GLAM reported redundant information via saliency maps in mammographic images where Radiologists A and B did not provide any annotations (see Fig. 1b,d (left breast which was cancer free) and Fig. 3a,c (right breast which was cancer free)), while GIMC did not mark the breast with a saliency map because it was cancer free. (see Figs. 2b,d, and 4a,c).

In a clinical situation like a national screening program, one radiologist might not mark a region as suspicious for malignancy, but the other may mark the region as a lesion while reading the same mammographic image independently. Figure 5a shows this scenario, where Radiologist B provided an annotation of the region with a larger green rectangle for Patient 3, but Radiologist A did not provide an annotation around the same region. Similarly, Radiologist A provided an annotation of the region with a red rectangle, but Radiologist B did not provide an annotation in Fig. 5c. Both GLAM and GMIC may agree with annotation from Radiologist B as shown in Figs. 5a and 6a. In addition, the two AI models provided saliency maps with obvious marks of suspicious lesions in Figs. 5c and 6c. There were 54 mammographic cases where the two radiologists disagreed about the location of lesions. Of these cases, the number of cases that GMIC and GLAM agreed with at least one of the radiologists is 47 and 45, respectively. From these two figures we can suggest that AI models can provide an effective method for decision making when double-reading independent radiologists do not agree with each other on the annotations of suspicious lesions.

Both results of GLAM and GMIC (see Fig. 7b,d) had an area that deviated from the overlapping area of two radiologists’ annotations, but the saliency maps of these two models with the addition of transfer learning (see Fig. 7a,c) encompassed more of the overlapping area and thus lead to more accurate results. For the GLAM with transfer learning in Fig. 7a, the AI was able to detect the same lesion area as seen within the green rectangle (i.e., annotation from Radiologist B), which was missed by GLAM only without transfer learning.

The lesion sensitivity of GLAM without and with transfer learning on the Dataset 2 was 71.2% and 86.5%, respectively. The lesion sensitivity of GMIC without and with transfer learning on the Dataset 2 was 72.3% and 89.7%, respectively. Both the GLAM and GMIC with transfer learning outperformed the AI models without transfer learning in correctly identifying the location of lesions.

### Sensitivity of GLAM and GMIC models on missed cases 

For “missed” cases, the GMIC model after transfer learning had a sensitivity of 82.98% while its sensitivity for the other two categories was 89.54% (*p*-value for the difference in sensitivity among three categories: 0.017). The GLAM model, transferred to our dataset, exhibited a sensitivity of 79.79% on “Missed” cases and a sensitivity of 87.25% on the remaining cases (*p*-value for the difference in among three categories: 0.006). After applying post-hoc pairwise comparison tests, we found significant differences in sensitivity between 'missed' and 'prior-vis' cancers, 'missed' and 'prior-invis' cancers, as well as 'prior-vis' and 'prior-invis' cancers for both the GLAM model with transfer learning (*p*-values < 0.01) and the GMIC model with transfer learning, with and without transfer learning (*p*-values < 0.01). The performance of the AI models was also evaluated in terms of sensitivity on mammograms with different groups of cancer sizes. The ANOVA test was conducted for statistical analysis of the AI models across different size groups of “prio-vis” and “prior-invis” groups. The sensitivity of GMIC models on Groups T1, T2 and T3 was 0.882, 0.928, and 0.947, respectively. The GMIC model (*p*-values < 0.05) demonstrated significant difference in sensitivity between each group of cancer sizes. The sensitivity of GLAM models on Groups T1, T2 and T3 was 0.851, 0.894, and 0.923, respectively. Similarly, GLAM (*p*-values < 0.05) demonstrated significant difference in sensitivity between each group. However, the sensitivity values of Group T1 were still higher than the sensitivity values observed for the “missed” category.

As stated, all cases in Dataset 1 were also included in Dataset 2 for concordance analysis. Table 1 shows the sensitivity of GMIC models with and without transfer learning for three categories of cancers: ‘missed’ cancers, ‘prior-vis’ cancers and ‘prior-invis’ cancers across four concordance levels in the dataset. As anticipated, the GMIC model with transfer learning provided the highest sensitivity of 94.1% in the ‘prior-vis’ group in the ‘almost perfect’ concordance level. Similarly, the sensitivity for the GLAM model with and without transfer learning was also shown in Table 1. The GMIC model outperformed the GLAM model in terms of the sensitivity in each of three categories of cancers. There were significant differences (*p*-values < 0.01) among four concordance levels in each cancer categories between the pre-trained and transfer learning modes of the two AI models.

**Table 1:** The sensitivity of GMIC and GLAM models with pre-trained and transfer learning modes for three categories of cancers in four concordance levels in Lifepool dataset.

Interpretation	Missed cancers		Prior vis cancers		Prior invis cancers	
	Pre-trained	Transfer learning	Pre-trained	Transfer learning	Pre-trained	Transfer learning
GMIC						
Almost perfect perfect perfect	72.6% (37)	86.3% (44)	84.3% (43)	94.1% (48)	81.1% (86)	92.5% (98)
Substantial	70.2% (33)	85.1% (40)	82.1% (32)	92.3% (36)	79.8% (83)	90.4% (94)
Moderate	66.7% (32)	81.3% (39)	80.0% (28)	88.6% (31)	77.3% (75)	87.6% (85)
Poor	64.3% (27)	78.6% (33)	76.7% (23)	85.3% (29)	74.7% (59)	84.8% (67)
GLAM						
Almost perfect	70.6% (36)	84.3% (43)	82.4% (42)	92.2% (47)	80.2% (85)	91.5% (97)
Substantial	68.1% (32)	83.0% (39)	79.4% (31)	89.7% (35)	76.9% (80)	87.5% (91)
Moderate	64.6% (31)	77.1% (37)	77.1% (27)	85.7% (30)	75.3% (73)	85.6% (83)
Poor	61.9% (26)	73.8% (31)	73.3% (22)	83.3% (25)	72.2% (57)	81.0% (64)

The number of correctly classified cases for each concordance level in each cancer category was shown inside the parentheses.

### Comparison on concordance classifications

As shown in Table 2, we compared the SIM and KLD between each saliency map of GLAM and GMIC with pre-trained mode in four interpretation levels of radiologists’ agreement on 856 abnormal mammograms, respectively. SIM between saliency maps of GLAM and GMIC was highest in ‘almost perfect’ (0.932), followed by ‘substantial’ (0.927), ‘moderate’ (0.912) and ‘poor’ (0.899). KLD between saliency maps of GLAM and GMIC was lowest in ‘almost perfect’ (0.153), followed by ‘substantial’ (0.166), ‘moderate’ (0.177) and ‘poor’ (0.190).

**Table 2:** Comparison of SIM and KLD between the saliency maps of two AI models with ‘pre-trained’ and ‘transfer learning’ modes in four concordance categories based on the annotations provided by two radiologists.

Interpretation	Saliency maps of GLAM and GMIC (with pre-trained mode)		Saliency maps of GLAM and GMIC (with transfer learning mode)	
	SIM	KLD	SIM	KLD
Almost perfect	0.932	0.153	0.961	0.097
Substantial	0.927	0.166	0.959	0.112
Moderate	0.912	0.177	0.955	0.128
Poor	0.899	0.190	0.947	0.143

As shown in Table 2, we also compared the SIM and KLD between the saliency maps of two AI methods with transfer learning mode on our dataset. The SIM between the saliency maps of the two models increased from 0.932 to 0.961 with transfer learning in the almost perfect category. The SIM values for two models with transfer learning also increased for all other concordance categories. KLD between saliency maps of two models decreased from 0.153 to 0.097 for two models with transfer learning in the ‘almost perfect’ category, with a similar trend in other categories.

### Specificity and sensitivity of GLAM and GMIC models on dataset 2

Table 3 shows the sensitivity of GLAM and GMIC models with and without transfer learning on four concordance levels in the image dataset. The pre-trained GLAM and GMIC models had the highest sensitivity of 82.4% and 83.7% in the ‘almost perfect’ cases, followed by the ‘substantial’, ‘moderate’, and ‘poor’ levels. Similarly, the transfer learning GLAM and GMIC models had the highest sensitivity of 93.2% and 95.5% in the ‘almost perfect’, followed by the ‘substantial’, ‘moderate’, and ‘poor’ levels. The GLAM and GMIC models had a specificity of 84.78% and 86.50%, which increased to 94.43% and 96.50% after transfer learning process. Applying an ANOVA test, both the pre-trained GLAM (*p*-value = 0.029) and GMIC (*p*-value = 0.014) models had statistically significant difference of sensitivity among four concordance levels. Similarly, both the transfer learning of GLAM (*p*-value = 0.021) and GMIC (*p*-value = 0.038) models showed statistically significant difference of sensitivity among four concordance levels. The GLAM and GMIC models with transfer learning demonstrated statistically significant improvement compared with pre-trained mode with an average sensitivity of 11.03% (*p*-value = 0.014) and 12.55% (*p*-value = 0.009) respectively among four concordance levels.

**Table 3:** Comparison of sensitivity of GLAM and GMIC models with (columns indicated as “Transfer”) and without (columns indicated as “Pre-trained”) transfer learning in four concordance levels of Dataset 2.

Concordance LEVEL	GLAM, Pre-TRAINED	GMIC, Pre-TRAINED	GLAM, TRANSFER	GMIC, TRANSFER
Almost Perfect	82.40%	83.70%	93.20%	95.50%
Substantial	81.00%	83.10%	92.00%	94.60%
Moderate	79.20%	80.50%	90.90%	93.40%
Poor	76.90%	76.80%	87.50%	90.80%
*p*-value	0.029	0.014	0.021	0.038

## Discussion

This study assessed the potential of AI tools to identify missed cancers retrospectively found in a dataset from a screening program with a double-reading practice, where each examination was independently interpreted by two readers. Our findings suggest that triple reading, involving two radiologists plus AI, could enhance the sensitivity of screening programs with two independent double readings. However, the results supported the need for further fine-tuning of the model when translating it from one setting with certain mammography machines and populations to another. Transfer learning resulted in significant improvements in both sensitivity and specificity values.

We also evaluated and compared the diagnostic accuracy of two AI breast cancer detection models developed for a mammogram database with different agreement levels of radiologists’ annotations (i.e., concordance). These four concordance levels also served as a metric indicating the difficulty of the cases. As anticipated, the results indicated that the stronger concordance between radiologists’ annotations, the higher the performance of GLAM and GMIC models. The SIM values from transfer learning models were greater than those from the pre-trained models in each concordance classification. In contrast, the KLD values obtained from the transfer learning models were less than those from the pre-trained models, considering that greater SIM values and lower KLD values reflect higher performance of the AI models. The “almost perfect” category had the highest SIM and lowest KLD values between saliency maps of the two transfer learning models, followed by “substantial”, “moderate”, and “poor” categories. Despite the differences in performance, for the best-performing model (i.e., the GMIC model) in all four concordance categories, the sensitivity values were above 90%. Therefore, even in the most challenging categories (i.e., poor and moderate), which are more demanding for radiologists and where the two AI tools agree with each other less, AI still exhibits a reasonable performance level.

The “missed” cancers refers to cases incorrectly classified as normal by two original radiologists during screening examinations. In a preliminary study^33^, the global radiomic signature features were extracted from “missed” cancers, “identified” cancers and “normal” cases. This global radiomic signature describes the overall appearance, texture, and density of mammographic examinations. In earlier studies, such a global signature was used to classify images into various breast density categories, estimating difficulty of case^34^ or to stratify the risk of breast cancer^35^. It was also associated with radiologists' initial impressions of the abnormality of a case^36^. The “identified” cancers were detected and biopsy-proven cancers and the immediate prior examination of the “missed” cancers were collected from the same database. AUCs of a random forest classifier on “missed” vs “identified” cancers, “identified” cancers vs “normal” cases, and “missed” cancers vs “normal” cases were 0.66, 0.65, and 0.53. The signatures were significantly different for “missed” vs “identified” cancers, “identified” cancers vs “normal” cases, but not for “missed” cancers vs “normal” cases. Hence, it is reasonable to assume these “missed” cases are among the most challenging cases as their overall mammographic texture resembles that of the “normal” cases. Upon evaluating the pre-trained GMIC, it was observed that the average sensitivity of “missed” cancers (68.5%) was notably lower than that of “prior-vis” (82.1%) and “prior-invis” cancers (78.2%). However, when employing GMIC with transfer learning, the average sensitivity of “missed” cancers significantly increased to 82.8%, closing the gap with “prior-vis” (90.1%) and “prior-invis” cancers (88.8%). Similar trends were observed with GLAM in both pre-trained and transfer learning modes, yielding significant results for the difference in sensitivity in two modes (*p*-values < 0.05). The performance of the AI models across the three categories—“prior-vis,” “prior-invis,” and “missed” cancers—suggests that, overall, the difficulty of cases when using AI models is comparable to the interpretation by radiologists. This implies that both AI models and breast radiologists may encounter challenges in interpreting particularly difficult mammograms, as seen in the “missed” category. However, the results also indicate that triple reading, involving the GMIC model and two radiologists, can significantly improve the detection of cancers currently missed in standard double reading practices. This approach may lead to the identification of over 82% of the cancers that would otherwise be overlooked. The findings underscore the potential of integrating AI models into the breast cancer screening process with double reading to enhance sensitivity and reduce the likelihood of missed diagnoses.

Cancer size could be considered as another surrogate variable for case difficulty. As anticipated, the diagnostic accuracy of AI systems is affected by the sizes of cancers in mammograms, with larger cancer sizes yielding more accurate cancer detection. However, the sensitivity values of AI tools for the smallest group were still higher than the sensitivity values observed for the 'missed' category. Therefore, being of a smaller size is not the only contributing factor that results in lower sensitivity for AI tools in the “missed” category. In a previous study^33^, global radiomic signature features were extracted from "missed" cancers, detected cancers, and normal cases. The findings revealed that the global radiomic signature of “missed” cancer cases closely resembled that of normal cases. This similarity suggests that interpreting “missed” cancer cases would be exceptionally challenging due to the overall global appearance of the images with radiomic signature features, potentially leading to false-negative diagnoses. The implication of this observation is significant and the reliance on the overall global image appearance might create a bias and contribute to radiologists mistakenly categorizing “missed” cancer cases as normal. To address this challenge, AI tools such as GMIC and GLAM, which excel in meticulous local image inspection, could potentially assist radiologists. These AI tools have the capacity to discern subtle local features that might be indicative of cancer even when the global radiomic signature suggests normalcy^33^. By leveraging the capabilities of AI models like GMIC and GLAM, radiologists could potentially overcome the limitations associated with global image interpretation. This, in turn, might enhance the accuracy of cancer detection by providing a more comprehensive and nuanced analysis that goes beyond the global radiomic signature.

In the current study, the normal cases were randomly selected from a pool of BSA archive, collected from multiple screening services across various jurisdictions within BreastScreen Australia. While our dataset inherently encompasses elements of benign disease and normal variants within the "normal cases" category, we recognize the importance of further enriching the dataset with a larger representation of benign cases from multicenter sources. It is expected that the false positive rate of GMIC and GLAM would be increased if the current study had specifically inserted benign cases. One of the main limitations of our study was the absence of cases that were specifically labelled as such, but rather, benign cases were included under a normal classification as BSA uses a recall/return to screening pathway. For transfer learning of the GLAM and GMIC models, we have only investigated the performance of the AI models from the point of view of malignancy detection and normal cases, which might include some benign mammographic features and normal variants. The Australian Screening dataset for this study did not have any labelled benign cases, so the results cannot comment on the GLAM and GMIC’s ability to identify discrete benign pathology that may require clinical action. Future research endeavors will focus on conducting larger multicenter studies to gather a more comprehensive dataset that includes a diverse range of benign conditions, thus enhancing the robustness and generalizability of AI models in mammography interpretation. Another limitation identified for the GLAM model was incorrectly localizing ‘false positive’ regions for cancers that were not present. As shown in Fig. 1b,d, the case was cancer free, yet the GLAM model returned malignancy features on mammographic images of LCC and LMLO. Similarly, the right breast was cancer free for Fig. 3a,c, but the GLAM model returned malignancy features on mammographic images of RCC and RMLO. One of the reasons for these incorrect decisions is that GLAM needs to use a larger number of patches selected from global saliency map for the local module, compared with the number of patches selected for GMIC model. Therefore, GLAM returned more obvious saliency maps of incorrect cancer locations than GMIC, as shown in Figs. 3a and 4a, and Figs. 1b and 2b.

In the clinical scenario of breast cancer detection via double-blinded reading in breast screening programs such as those in Australian^37^, two independent radiologists may sometimes provide different reading outcomes on the same images. Two methods can be used to address discordant reads. One is to use a third radiologist who is a highly experienced breast reader to arbitrate but this can be time consuming and expensive. The other method arranges the two original radiologists to discuss the two differing interpretation and agree on the outcome as a single recommendation. Our research shows promise for the use of GLAM and GMIC models to provide cancer detection results in situations of discordant outcomes, for example in the blue saliency maps in Figs. 5c and 6c, where the only Radiologist A and the AI models identified a cancer. Triple reading, including GMIC and two independent radiologists, might provide an effective way to improve the detection rate of cancers in the standard double reading breast screening program because the model successfully identified > 80% of previously missed cancer cases.

The GMIC and the GLAM models performed well on all four interpretation levels of concordance in our dataset which consists of mammography exams from different manufacturers such as Sectra, Fuji, Siemens, Hologic, GE Healthcare and Philips Healthcare. While many existing AI modes demonstrate good performance on mammographic images from a sole manufacturer (e.g., Ribli et al.’s^38^ paper with Hologic, Dhungel et al.’s paper^39^ with Siemens, and Yang et al.’s paper^40^ with Siemens), our study applied the GLAM and GMIC to a range of vendor images and found high SIM values (> 0.91) between the saliency maps of the AI models and radiologists’ annotations regardless of the image source. The results showed that the GMIC model was successful in finding mammographic cases at specificity and sensitivity of > 90% for cases with different manufacturers.

## Conclusions

In this paper, we presented diagnostic accuracy of the two AI models in interpreting mammographic images based on the strength of concordance between radiologists in breast cancer detection. The study also supports the potential of AI tools to identify missing cancers within a double-reading practice, where each examination was independently interpreted by two readers. The results showed that AI systems testing on mammographic images with stronger concordance data from radiologists outperformed that with weaker concordance levels. Transfer learning for the pre-trained AI models can also improve the performance, which suggests that AI models can be used as an effective method for decision making when radiologists do not agree with each other in the annotations of suspicious lesions. The average sensitivity of AI models across different groups of cancer sizes outperformed that of the “missed” cancers, indicating that cancer sizes is not the only factor affecting the AI performance on screening mammograms.

For future work, we will conduct eye tracking experiments with radiologists and breast physicians on of the “missed” cancers, “prior-vis” cancers and “prior-invis” cancers. We intend to compare heat maps of radiologists with the saliency maps of AI models on these three categories.

## Data Availability

The data supporting this study’s findings are available on request from the corresponding authors.
